# Diabetic Self-Care Practices and Quality of Life Among Diabetic Patients in Qassim Region, Saudi Arabia: A Cross-Sectional Study

**DOI:** 10.7759/cureus.110932

**Published:** 2026-06-15

**Authors:** Asayel S Almutairi, Saulat Jahan

**Affiliations:** 1 Family Medicine, Family Medicine Academy, Qassim Health Cluster, Buraidah, SAU; 2 Epidemiology and Public Health, Family Medicine Academy, Qassim Health Cluster, Buraidah, SAU

**Keywords:** qassim, quality of life, saudi arabia, self care practices, type 2 diabets mellitus

## Abstract

Introduction: Diabetes is a common chronic disease that requires effective self-care practices. These practices play an important role in improving patients’ quality of life (QoL). This study aimed to determine the level of diabetic self-care practices and QoL of diabetic patients in the Qassim region, Saudi Arabia. Additionally, it explored the factors influencing the QoL of these patients.

Methods: A cross-sectional study was conducted among diabetic patients in primary health care centers. Data were collected using an Arabic self-administered electronic questionnaire, including the modified validated Arabic version of the Summary of Diabetes Self-Care Activities (SDSCA) and the modified validated Arabic version of the Diabetes Quality of Life (DQoL) questionnaire. The questionnaire was pilot tested prior to data collection. Data were analyzed using IBM SPSS Statistics for Windows, Version 29 (Released 2022; IBM Corp., Armonk, New York).

Results: A total of 155 participants were included in the study. The mean age of the study participants was 53 ± 10.8 years, with almost equal representation of males (49.7%) and females (50.3%). Most participants (78.1%) were treated with oral medications in the form of tablets, and less than one-third (29.0%) had HbA1c levels below 7%. Medication adherence showed the highest self-care scores (6.3 ± 1.73 days per week), followed by glucose monitoring (3.4 ± 2.42 days per week). QoL was significantly associated with gender and HbA1c levels. Positive correlations were found between QoL and both medication adherence and shoe checking practices.

Conclusion: Medication adherence was the most commonly practiced self-care behavior and was associated with better QoL. However, lifestyle-related practices were suboptimal. These findings highlight the need for targeted interventions focusing on lifestyle modification to improve overall diabetes management and patient outcomes.

## Introduction

Diabetes mellitus is a collection of autoimmune, metabolic, and genetic illnesses that have the characteristic feature of hyperglycemia [1]. Diabetes' chronic consequences are classified as microvascular or macrovascular, with the former being significantly more common than the latter. Microvascular problems include neuropathy, nephropathy, and retinopathy, whereas macrovascular issues include heart disease, stroke, and peripheral artery disease (PAD). Diabetic foot syndrome is characterized by foot ulcers linked with neuropathy, PAD, and infection. It is a primary cause of lower limb amputation [2].

Individuals with diabetes require self-care that goes beyond blood glucose control and includes preventing impairments and consequences [3]. Several important self-care habits are associated with positive outcomes, including healthy food, physical activity, blood glucose monitoring, and medication adherence [4]. Individuals with diabetes who adopt self-care practices report improved glucose control, fewer complications, and a higher quality of life (QoL) [5]. They have been found to have a significant impact on the progression and development of diabetes by managing their own care [4]. Social support, particularly from family members and spouses, plays a crucial role in promoting self-care behaviors among diabetes patients [6]. The 2016 Standards of Medical Care in Diabetes stress a patient-centered, customized approach to diabetes management. Care should be customized in accordance with the patient’s age, comorbidities, and lifestyle preferences. A multidisciplinary team of primary care clinicians, specialists, and other health professionals, such as nutritionists and mental health counsellors, is essential for providing comprehensive care [7].

The objectives of the study were to evaluate the level of diabetic self-care practices among diabetic patients in the Qassim region using the modified validated Arabic version of the Summary of Diabetes Self-Care Activities (SDSCA) questionnaire and to assess the QoL among diabetic patients using the modified validated Arabic version of the Diabetes Quality of Life (DQoL) questionnaire. Furthermore, the study also identified sociodemographic and clinical factors associated with self-care practices and QoL and assessed the relationship between diabetic self-care practices and QoL among diabetic patients in the Qassim region, Saudi Arabia.

## Materials and methods

Study design and setting

This study employed a cross-sectional design to assess the relationship between diabetes self-care practices and QoL. Six primary healthcare centers in Buraidah, Qassim region, Saudi Arabia, were selected through convenience sampling.

Sample size calculation

The sample size was calculated using OpenEpi (Atlanta, Georgia), a widely used tool for epidemiological studies. To determine the sample size, a study conducted in Najran, Saudi Arabia, was referenced, which found that 90.1% of patients with diabetes exhibited poor self-care behaviors, as measured by the Diabetes Self-Management Questionnaire (DSMQ) [8]. Using this prevalence rate and a 95% confidence level, the estimated sample size was 138 participants. However, the sample size was increased to 155 participants.

Sampling technique

Participants were recruited consecutively from diabetic clinics in primary healthcare centers during their routine follow-up visits. Research assistants were stationed at these clinics during working hours to approach eligible patients after their appointments. This on-site recruitment continued until the required sample size was achieved for each center. If a selected patient declined participation, the next eligible patient visiting the clinic was approached.

Selection criteria

Inclusion Criteria

The study population comprised Saudi adults aged 18 years and above with diagnosed type 2 diabetes mellitus who were ambulatory and capable of providing informed consent.

Exclusion criteria

Patients with cognitive impairment that could affect their ability to complete questionnaires accurately, pregnant women with gestational diabetes, those with severe psychiatric disorders affecting self-care abilities, non-Saudi residents, patients diagnosed with diabetes for less than six months, and individuals unable to communicate effectively in Arabic.

Data collection

Data were collected using a structured questionnaire consisting of three sections: sociodemographic and clinical information; a modified validated Arabic version of the SDSCA questionnaire [9], originally developed by Toobert et al. [10]; and a modified validated Arabic version of the DQoL questionnaire [11], originally developed by the DCCT research group [12]. The sociodemographic section included age, gender, education level, marital status, employment status, and monthly income. Clinical data included duration of diabetes, type of treatment, presence of complications, and latest HbA1c readings. Some items from both questionnaires were modified or removed to improve clarity and cultural suitability for the study population. The modified questionnaire was pilot tested on 10 participants prior to data collection to ensure clarity and feasibility. No substantial modifications were required following the pilot testing. Data collection was conducted using an Arabic self-administered electronic questionnaire completed by the participants. Data were collected from December 2025 to February 2026.

Data analysis

Statistical analyses were performed using IBM SPSS Statistics for Windows, Version 29 (Released 2022; IBM Corp., Armonk, New York). For scoring calculations, the SDSCA scores were computed by averaging the number of days (0-7) for each self-care domain (diet, exercise, blood glucose testing, and foot care), where higher scores indicated better self-care practices. The DQoL scores were calculated using a 5-point Likert scale (1-5), with mean scores computed for each domain. Higher scores indicated better QoL. Internal consistency of the questionnaire was assessed using Cronbach’s alpha coefficient, with 0.70 considered the minimum acceptable level of reliability. Descriptive statistics were used to summarize demographic characteristics using frequencies, percentages, means, and standard deviations. The relationship between self-care practices and QoL scores was examined using the Pearson correlation coefficient. Comparisons across different demographic and clinical groups were analyzed using appropriate statistical tests, with statistical significance set at p < 0.05.

Ethical considerations

Ethical approval was obtained from the Qassim Regional Research Ethics Committee (approval number: 607-46-0150). Verbal informed consent was obtained from all participants after explaining the study objectives and procedures. Participation was voluntary, and participants had the right to withdraw at any time. All collected data were coded and kept confidential, accessible only to the research team. The study was conducted in accordance with the Declaration of Helsinki for medical research involving human subjects.

## Results

A total of 155 diabetic patients participated in this study. The mean age of study participants was 53 ± 10.8 years. Table 1 displays the demographic characteristics of the study participants. The male (n = 77, 49.7%) and female (n = 78, 50.3%) representation was almost equal. Regarding education, 38 participants (24.5%) had a bachelor's degree, and eight participants (5.2%) had received a postgraduate degree.

**Table 1:** Socio-demographic characteristics of the study participants (n = 155). SAR: Saudi Arabian Riyal.

Demographic characteristics		Number	Percentage
Gender	Male	77	49.7%
	Female	78	50.3%
Marital status	Unmarried	11	7.1%
	Married	120	77.4%
	Divorced	11	7.1%
	Widowed	13	8.4%
Education level	No formal education	18	11.6%
	Primary school	19	12.3%
	Middle school	16	10.3%
	High school	31	20.0%
	Technical degree	25	16.1%
	Bachelor	38	24.5%
	Postgraduate degree	8	5.2%
Employment status	Unemployed	50	32.3%
	Employed	75	48.4%
	Retired	27	17.4%
	Other	3	1.9%
Monthly income (SAR)	Less than 5,000	43	27.7%
	5000-10000	66	42.6%
	10,001-20,000	41	26.5%
	More than 20,000	5	3.2%

Table 2 presents the diabetes-related characteristics of the study participants. A total of 121 (78.1%) were treated with oral medications in the form of tablets, while 34 (21.9%) were using both tablets and insulin. Regarding glycemic control, more than half of the participants (n = 84, 54.2%) had an HbA1c level between 7% and 9%, while 45 (29.0%) had HbA1c levels below 7%. Comorbidities were reported in 60 (38.7%) of participants. The mean duration of diabetes was 8.39 ± 7.5 years.

**Table 2:** Diabetes-related characteristics of the study participants (n = 155). HbA1c: hemoglobin A1c; SD: standard deviation.

Diabetes-related characteristics		Number	Percentage
Diabetes treatment	Tablet	121	78.1%
	Insulin	0	0.0%
	Tablet and insulin both	34	21.9%
Last HbA1c level	Less than 7%	45	29.0%
	7-9%	84	54.2%
	More than 9%	21	13.5%
	I don’t know	5	3.2%
Presence of comorbidities	No	95	61.3%
	Yes	60	38.7%
Duration of diabetes (years) (mean ± SD)		8.39 ± 7.5 years	

Table 3 summarizes diabetes self-care activities during the previous seven days. The highest mean and median scores were observed for medication adherence (mean = 6.3 ± 1.73; median = 7 days), indicating high adherence to prescribed medications. Diet-related self-care showed a median of four days, while blood glucose testing, exercise, foot care, and shoe inspection had median values of approximately two days during the previous week.

**Table 3:** SDSCA during the last seven days (n = 155). SDSCA: Summary of Diabetes Self-Care Activities; SD: standard deviation; IQR: interquartile range.

Diabetes self-care activity	Mean ± SD	Median (IQR)	Minimum	Maximum
Number of days tested for blood glucose	3.4 ± 2.42	2 (1-4)	0	7
Number of days followed the recommended diet	2.5 ± 2.00	4 (1-5)	0	7
Number of days exercised for at least 30 minutes	2.3 ± 2.13	2 (1-4)	0	7
Number of days checked and cared for feet	3.0 ± 2.54	2 (1-5)	0	7
Number of days inspected the inside of shoes	2.3 ± 2.33	2 (0-4)	0	7
Number of days took diabetes medications as prescribed	6.3 ± 1.73	7 (7-7)	0	7

Reliability testing of the adapted DQoL scale revealed high internal consistency (Cronbach's α = 0.88).

Table 4 presents the satisfaction domain of the DQoL questionnaire. Most participants reported being satisfied or very satisfied with their current diabetes treatment, with 68 (43.9%) and 61 (39.4%) participants, respectively. Similarly, 69 (44.5%) participants were satisfied and 55 (35.5%) were very satisfied with their knowledge about diabetes. Regarding general life satisfaction, the majority of participants (n = 94, 60.6%) reported being very satisfied.

**Table 4:** Satisfaction domain scores of the DQoL instrument (n = 155). DQoL: Diabetes Quality of Life.

Satisfaction domain (DQoL)		Number	Percentage
How satisfied are you with your current treatment?	Very dissatisfied	3	1.9%
	Dissatisfied	7	4.5%
	Neutral	16	10.3%
	Satisfied	68	43.9%
	Very Satisfied	61	39.4%
How satisfied are you with the flexibility you have in your diet?	Very dissatisfied	2	1.3%
	Dissatisfied	11	7.1%
	Neutral	34	21.9%
	Satisfied	70	45.2%
	Very satisfied	38	24.5%
How satisfied are you with the burden your diabetes is placing on your family?	Very dissatisfied	6	3.9%
	Dissatisfied	7	4.5%
	Neutral	30	19.4%
	Satisfied	59	38.1%
	Very satisfied	53	34.2%
How satisfied are you with your knowledge about your diabetes?	Very dissatisfied	3	1.9%
	Dissatisfied	11	7.1%
	Neutral	17	11.0%
	Satisfied	69	44.5%
	Very satisfied	55	35.5%
How satisfied are you with life in general?	Very dissatisfied	1	0.6%
	Dissatisfied	4	2.6%
	Neutral	14	9.0%
	Satisfied	42	27.1%
	Very satisfied	94	60.6%

Table 5 presents the impact domain of the DQoL questionnaire. Most participants reported that diabetes never interfered with their family life (n = 95, 61.3%) or social relationships (n = 88, 56.8%). Similarly, the majority indicated that diabetes never interfered with exercise (n = 101, 65.2%) or caused them to miss work, school, or household duties (n = 103, 66.5%). However, 59 (38.1%) participants reported occasionally feeling physically unwell because of diabetes, while 52 (33.5%) reported feeling restricted by their diet.

**Table 5:** Impact domain scores of the DQoL instrument (n = 155). DQoL: Diabetes Quality of Life.

Impact domain (DQoL)		Number	Percentage
How often do you have low blood sugar?	Never	45	29.0%
	Very rarely	65	41.9%
	Sometimes	39	25.2%
	Often	6	3.9%
	Always	0	0.0%
How often do you feel physically ill because of diabetes?	Never	54	34.8%
	Very rarely	30	19.4%
	Sometimes	59	38.1%
	Often	9	5.8%
	Always	3	1.9%
How often does your diabetes interfere with your family life?	Never	95	61.3%
	Very rarely	37	23.9%
	Sometimes	16	10.3%
	Often	7	4.5%
	Always	0	0.0%
How often does your diabetes limit your social relationships and friendships?	Never	88	56.8%
	Very rarely	44	28.4%
	Sometimes	18	11.6%
	Often	5	3.2%
	Always	0	0.0%
How often do you feel restricted by your diet?	Never	36	23.2%
	Very rarely	47	30.3%
	Sometimes	52	33.5%
	Often	18	11.6%
	Always	2	1.3%
How often does your diabetes interfere with your exercising?	Never	101	65.2%
	Very rarely	35	22.6%
	Sometimes	14	9.0%
	Often	4	2.6%
	Always	1	0.6%
How often do you miss work, school, or household duties because of your diabetes?	Never	103	66.5%
	Very rarely	33	21.3%
	Sometimes	16	10.3%
	Often	3	1.9%
	Always	0	0.0%

Table 6 presents the worry domain of the DQoL questionnaire. Most participants reported that they never worried about losing consciousness due to diabetes (n = 95, 61.3%). However, a considerable proportion of participants reported sometimes worrying about developing diabetes-related complications (n = 48, 31.0%). Additionally, 44 (28.4%) of participants reported that they never worried about developing complications from diabetes.

**Table 6:** Worry domain scores of the DQoL instrument (n = 155) DQoL: Diabetes Quality of Life.

Worry domain (DQoL)		Number	Percentage
How often do you worry about losing consciousness due to your diabetes?	Never	95	61.3%
	Very rarely	29	18.7%
	Sometimes	28	18.1%
	Often	2	1.3%
	Always	1	0.6%
How often do you worry that you will get complications from your diabetes?	Never	44	28.4%
	Very rarely	39	25.2%
	Sometimes	48	31.0%
	Often	18	11.6%
	Always	6	3.9%

Table 7 displays the association of sociodemographic and clinical characteristics with QoL among study participants. A significant difference in QoL scores was observed between males and females (p = 0.018), with males having a higher mean rank. HbA1c levels were also significantly associated with QoL (p = 0.011), where participants with HbA1c < 7% had the highest QoL mean rank. No statistically significant differences in QoL were observed across age groups, marital status, education level, employment status, monthly income, or presence of comorbidities.

**Table 7:** Association of sociodemographic and clinical characteristics with QoL among study participants ¶ The Mann-Whitney U test was used for comparison between two groups. ¥ The Kruskal-Wallis test was used for comparison between more than two groups. QoL: quality of life; HbA1c: hemoglobin A1c.

Variable	Category	N	Mean Rank	p-value
Age group	<45 years	44	73.66	0.428¥
	46-65 years	87	77.36	
	>65 years	24	88.29	
Gender	Male	77	86.61	0.018¶
	Female	78	69.50	
Marital status	Unmarried	11	63.73	0.062¥
	Married	120	82.65	
	Divorced	11	48.73	
	Widowed	13	71.92	
Education level	No formal education	18	73.44	0.880¥
	Primary school	19	79.21	
	Middle school	16	75.69	
	High school	31	81.06	
	Technical degree	25	86.38	
	Bachelor	38	70.99	
	Higher education	8	85.25	
Employment status	Unemployed	50	74.89	0.083¥
	Employed	75	72.57	
	Retired	27	95.96	
	Other	3	103.83	
Monthly income	<5,000	43	70.09	0.322¥
	5,000-10,000	66	80.04	
	10,001-20,000	41	79.59	
	>20,000	5	106.10	
HbA1c level	<7%	45	89.04	0.011¥
	7-9%	84	73.24	
	>9%	21	55.50	
Comorbidities	No	95	80.23	0.437¶
	Yes	60	74.48	

We analyzed the association of demographic and clinical characteristics with diabetes self-care practices among study participants. Exercise practices differed significantly according to gender (p = 0.013), marital status (p = 0.003), education level (p = 0.002), employment status (p = 0.006), monthly income (p = 0.004), and HbA1c level (p < 0.001). Dietary practices were significantly associated with employment status (p = 0.012), monthly income (p = 0.041), and HbA1c level (p = 0.001).

On examining the correlation between diabetes self-care practice and QoL among the study participants, no statistically significant correlations were observed between QoL and diet adherence, exercise, blood glucose testing, or foot care practices. However, QoL was positively associated with medication adherence (r = 0.286, p < 0.001).

## Discussion

Our study found that medication adherence was the most frequently practiced self-care activity among participants. In contrast, other self-care behaviors such as exercise, blood glucose testing, foot care, and shoe inspection were relatively low. A previous study from Saudi Arabia concluded that despite satisfactory adherence to prescribed medications, self-care management practices among individuals with type 2 diabetes in Saudi Arabia require significant improvement [13].

The high level of medication adherence observed in the present study may indicate that patients recognize the importance of pharmacological treatment in controlling diabetes, as it is a consistent finding in other studies. A study conducted in Jeddah reported that medication adherence was the most commonly practiced diabetes self-care activity among patients attending primary health care centers, while lifestyle-related practices such as physical activity were less frequently practiced [14]. A cross-sectional study of diabetic patients at the Sultan Bin Abdul-Aziz Humanitarian City found that foot care was the most commonly reported self-care practice, followed by medication self-management. Exercise self-management and blood sugar testing were the two least frequently mentioned practices [15].

Internationally, similar trends have been observed. A systematic review by Mogre et al. reported that adherence to medication was generally higher than adherence to lifestyle-related practices such as diet and exercise in low- and middle-income countries [16]. Furthermore, Ketema et al. found that adherence to recommended diabetes self-care practices among patients in Ethiopia was generally suboptimal, particularly for lifestyle-related behaviors [17]. A systematic analysis of diabetes self-care habits across 27 low- and middle-income countries showed consistent findings. The lowest adherence was in foot care, practiced as little as 2.2 days per week, with a minimum adherence rate of 17.0%. The highest adherence was in medication adherence, practiced up to 6.8 days per week, with a maximum adherence rate of 97.0% [16]. A meta-analysis of studies in Ethiopia on diabetes self-care practices found the highest adherence in diabetic foot care at 58%, while the lowest adherence was in self-monitoring of blood glucose at 28% [17].

Studies conducted in Saudi Arabia, including those from Riyadh and Najran, reported findings similar to our study that self-care practices were suboptimal, particularly for lifestyle-related behaviors [8,13]. The relatively low levels of lifestyle-related practices such as exercise and regular blood glucose monitoring may reflect challenges in maintaining long-term behavioural changes. These findings suggest that although patients may follow medical treatment, they may face barriers in implementing lifestyle modifications. These barriers may include lack of awareness, limited health education, or difficulties in sustaining healthy lifestyle habits over time. Additionally, cultural factors, including religious beliefs, may influence patients’ perceptions of their QoL and contribute to better coping and acceptance of living with a chronic condition.

In our study, most participants reported moderate to high levels of satisfaction with their QoL. QoL was significantly associated with gender and HbA1c levels. Moreover, medication adherence showed a significant positive correlation with QoL. Similar findings were reported in a study conducted in the Hail region of Saudi Arabia, which found that many diabetic patients reported acceptable levels of QoL despite living with a chronic condition [18]. A study on health-related quality of life (HRQoL) among type 2 diabetes patients in Riyadh found that the average HRQoL score was 0.71, with males having a higher score (0.74) than females (0.58) [19].

The findings of this study highlight the importance of strengthening diabetes self-management programs. Healthcare providers should emphasize comprehensive diabetes care, including lifestyle modifications such as regular physical activity, blood glucose monitoring, and foot care. Structured diabetes education programs may also improve patients’ knowledge and self-management behaviors. The Diabetes Education and Self-Management for Ongoing and Newly Diagnosed (DESMOND) program demonstrated that structured diabetes education can significantly improve diabetes self-management behaviors and patient outcomes [20].

This study has several limitations. First, the cross-sectional design limits the ability to establish causal relationships between diabetes self-care practices and QoL. Second, the study relied on self-reported questionnaires, which may introduce recall bias, as participants may not accurately remember their self-care activities, and social desirability bias, as participants may report more favorable health behaviors. Third, selection bias may have occurred because participants were recruited from primary healthcare centers, potentially excluding diabetic patients who do not regularly attend follow-up visits. Additionally, HbA1c values were based on participants’ self-reported latest readings and were not objectively verified through medical records or laboratory measurements. Finally, the study was conducted in a single region, which may limit the generalizability of the findings to other populations.

## Conclusions

This study found that medication adherence was the most frequently practiced diabetes self-care activity among participants. At the same time, lifestyle-related behaviors such as exercise and blood glucose monitoring were less commonly practiced. QoL was generally moderate to high among participants and was significantly associated with gender and HbA1c levels. These findings highlight the importance of improving lifestyle-related self-care behaviors to enhance the QoL among diabetic patients. Future studies should include larger and more diverse populations to further investigate the relationship between diabetes self-care practices and QoL. Longitudinal studies are recommended to assess the long-term impact of self-care behaviors on health outcomes. Qualitative research may also provide deeper insights into patients’ experiences, perceptions, and barriers related to diabetes self-management. Further research is needed to explore factors associated with QoL and determinants of glycemic control, particularly those influencing HbA1c levels. In addition, healthcare systems should focus on implementing structured diabetes education programs and lifestyle interventions, particularly those targeting physical activity and blood glucose monitoring, to enhance self-care practices and improve overall patient outcomes.
